# Supplementary material for: ER-associated ubiquitin ligase HRD1 programs liver metabolism by targeting multiple metabolic enzymes
Source: Nat Commun. 2018 Sep 10;9:3659. doi: 10.1038/s41467-018-06091-7 (PMC6131148; doi:10.1038/s41467-018-06091-7)
Supplement: Supplementary file 1 — Supplementary Information [file 41467_2018_6091_MOESM1_ESM.pdf]

Supplementary Information for

ER-associated ubiquitin ligase HRD1 programs liver metabolism by targeting multiple metabolic enzymes

Wei et al.

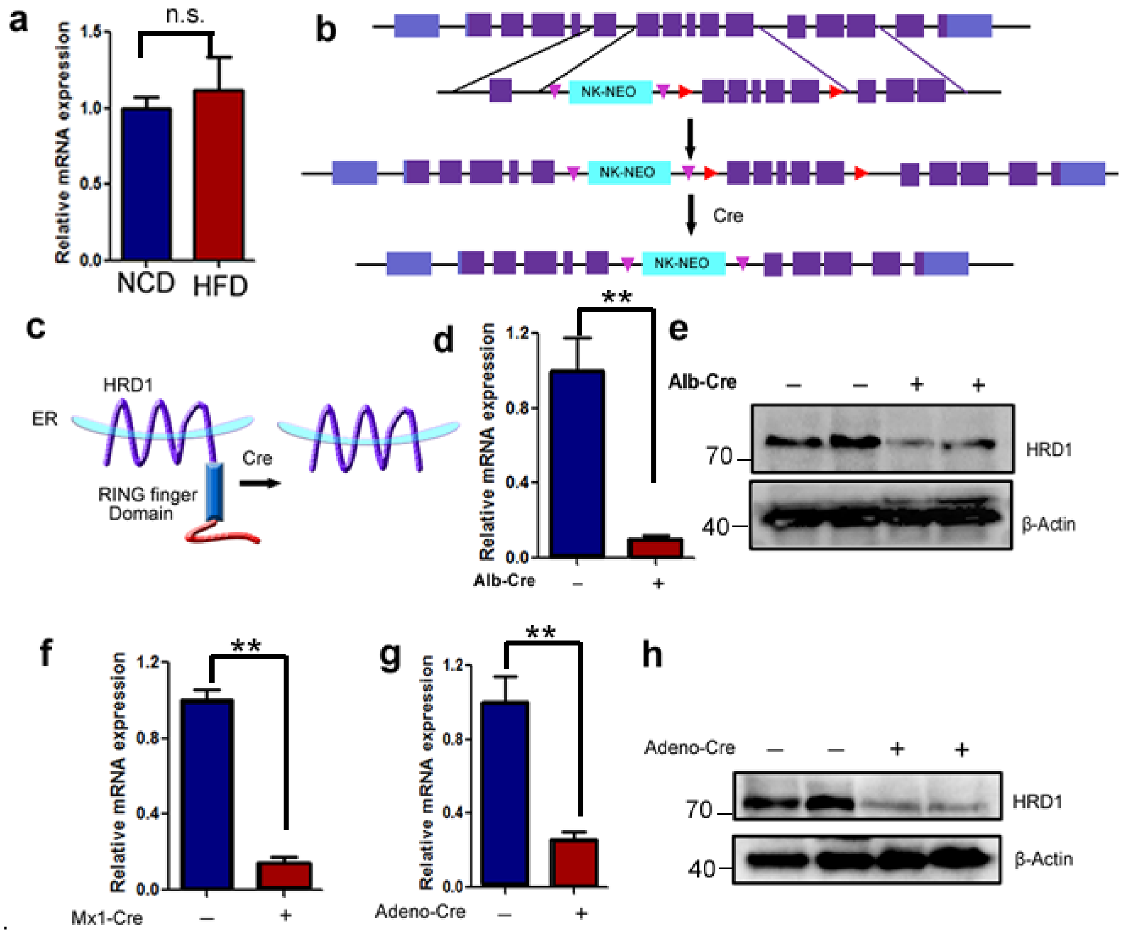

Supplementary Fig. 1 Hepatocye-specific *Hrd1* Condition Knockout mice generation. (**a**) Relative mRNA levels in a normal chow or after 14 weeks High fat diet treatment. (n=5 for each group) (**b**) Structures of the *Hrd1* WT and targeted alleles. (**c**) Domain structure of Hrd1 protein. (**d&e**) Hepatic HRD1 mRNA (**d**) and protein levels (**e**) from WT and *LKO* mice. (**f**) Hepatic *Hrd1* mRNA 5 days after Poly (I:C) injection. (n=5 for each group). (**g&h**) hepatic HRD1 mRNA (**g**) and protein levels (**h**) after Adeno-Cre virus injection (n=5 for each group). The data are representative of three independent experiments (mean ± s.d.). *: $P<0.05$. **: $P<0.01$ by unpaired student's t test.

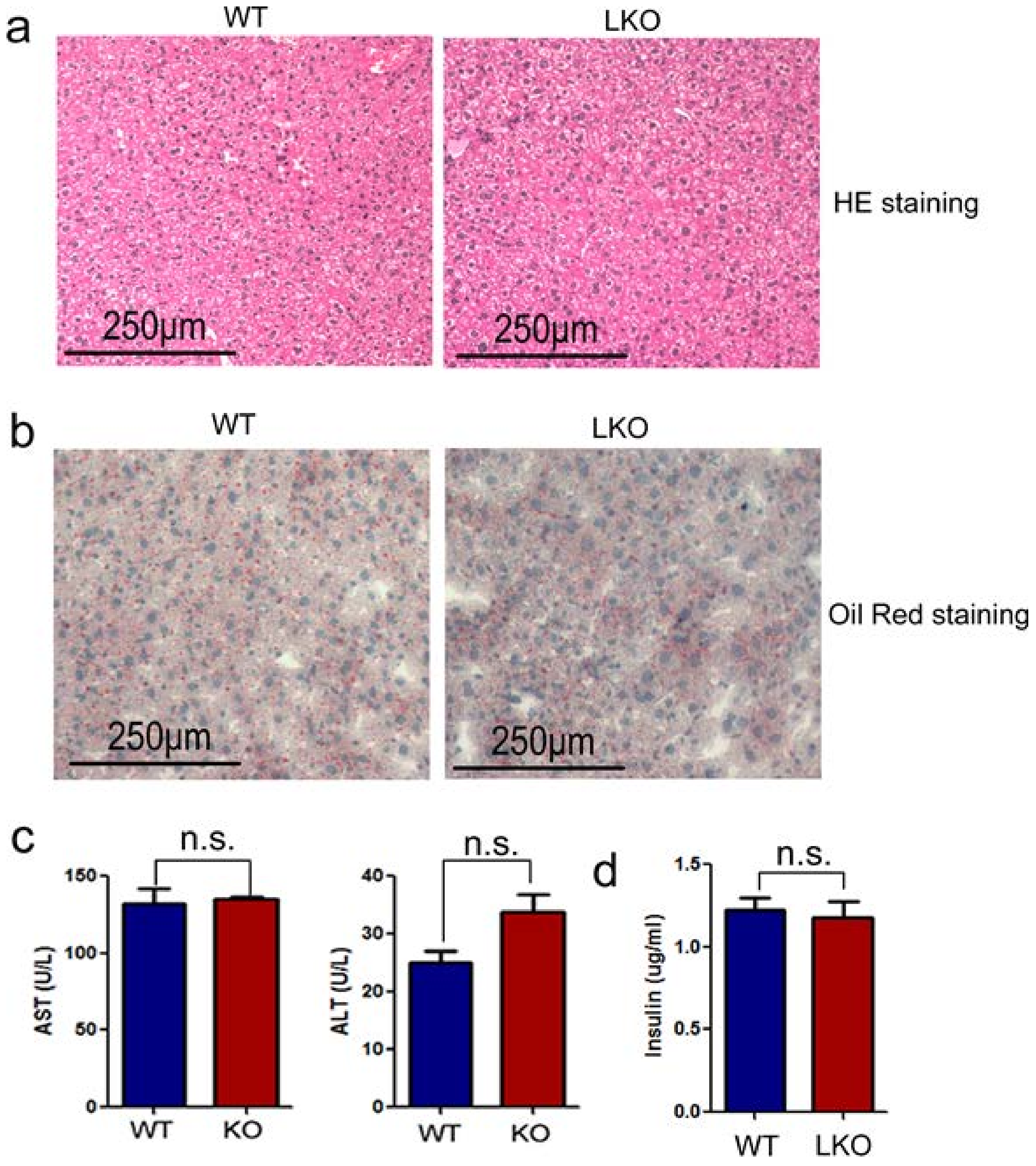

Supplementary Fig. 2 HRD1 deletion didn't result in liver toxicity. (a) haematoxylin and eosin (HE) staining of liver from WT and LKO mice fed with normal chow diet. **(b)** Oil red staining of liver from WT and LKO mice fed with normal chow diet. **(c)** AST and ALT were measured in the serum of WT and HRD1 LKO mice in a refed condition. (n=5 for each group) **(d)** Insulin levels were measured in the serum of WT and HRD1 LKO mice in a refed condition. (n=5 for each group). The data are representative of three independent experiments (mean ± s.d.). *: P<0.05. **: P<0.01 by unpaired student's t test.

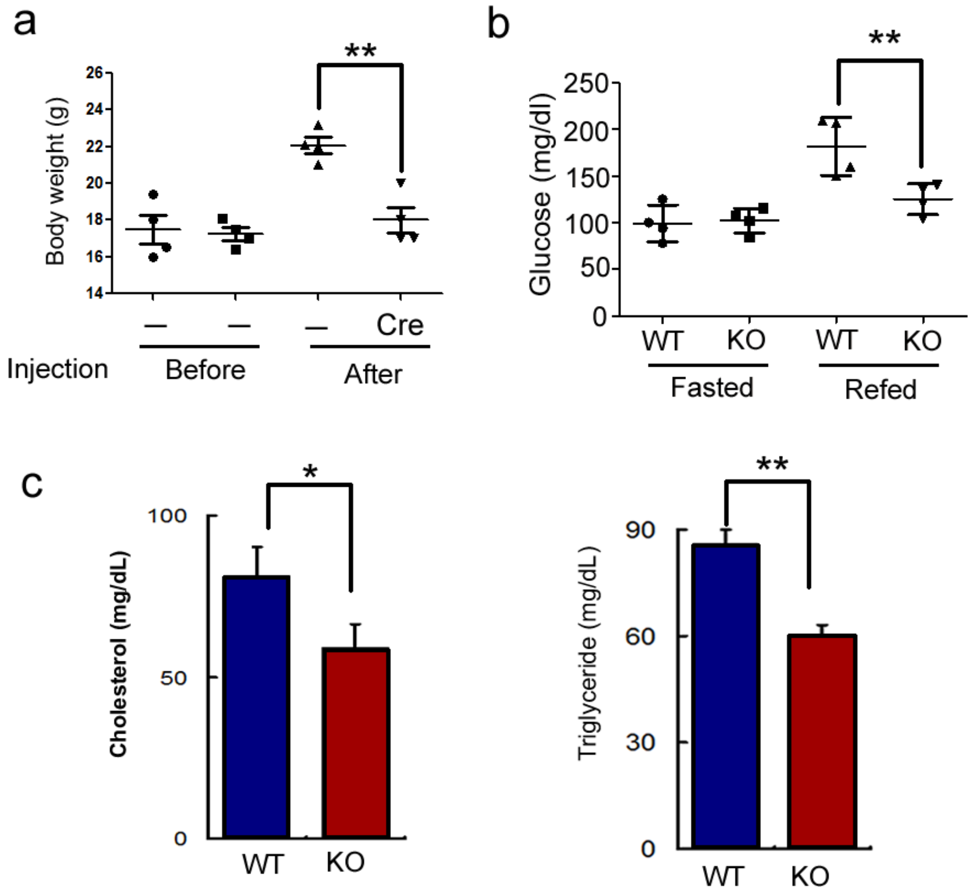

Supplementary Fig. 3 HRD1 deletion induced by Cre-Adenovirus virus injection will decrease body weight gain, glucose and lipid levels (**a**) The body weight gains of the *Hrd1*$^{F/F}$ before and 1 month after Adeno-Cre virus Injection. (n=4 for each group). (**b**) Blood glucose was measured 5 days after Adeno-Cre virus injection in the fasted and refed condition. (n=4 for each group). (**c**) Serum cholesterol and TG level were measured 5 days after Adeno-Cre virus injection. (n=6 for each group). The data are representative of three independent experiments (mean±SEM) *: $P<0.05$. **: $P<0.01$ by unpaired student's t test.

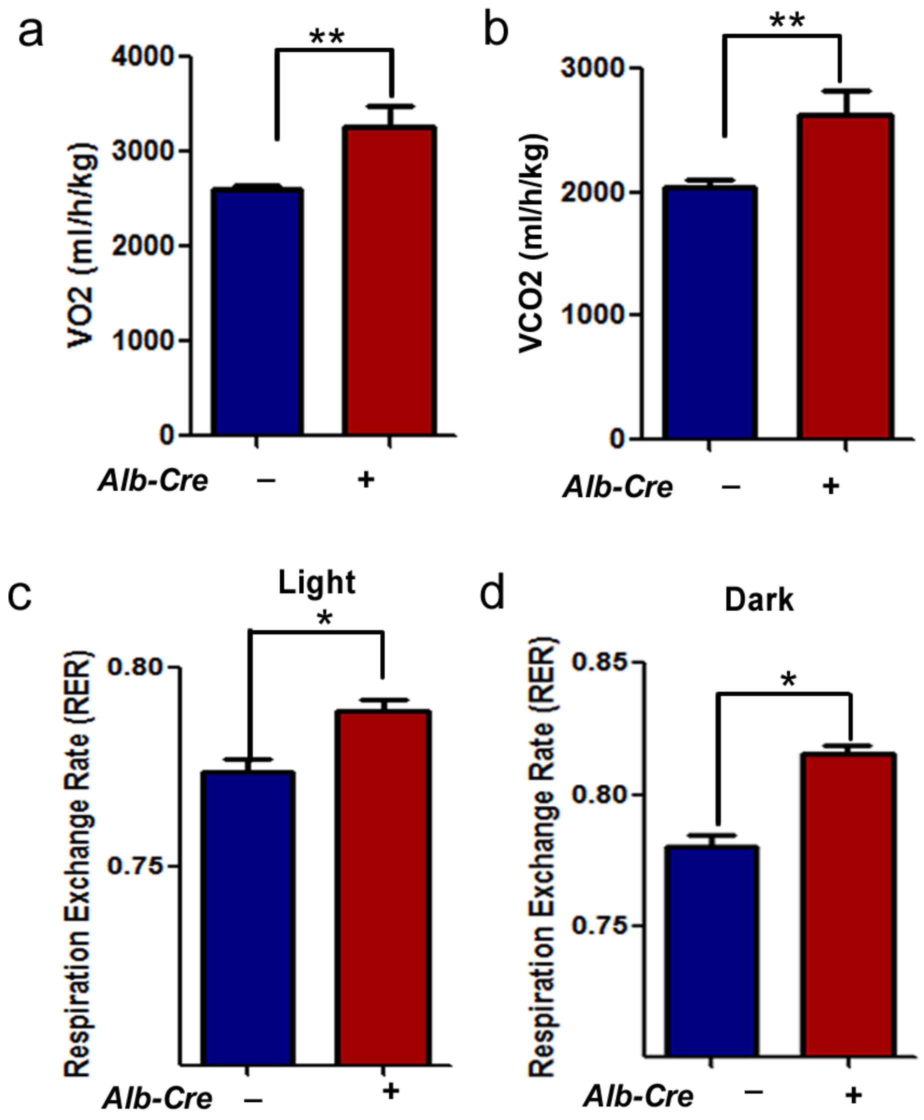

Supplementary Fig. 4 Hepatic HRD1 deletion increased the energy expenditure. (**a&b**) O2 consumption (**a**) and CO2 (**b**) Production of WT and *L-Hrd1-/-* mice after 14 weeks HFD treatment. (n=5 for each group). (**c&d**) RER of WT and *L-Hrd1-/-* mice after 14 weeks HFD treatment. (n=5 for each group). The data are representative of three independent experiments (mean±SEM) *: $P<0.05$. **: $P<0.01$ by unpaired student's t test.

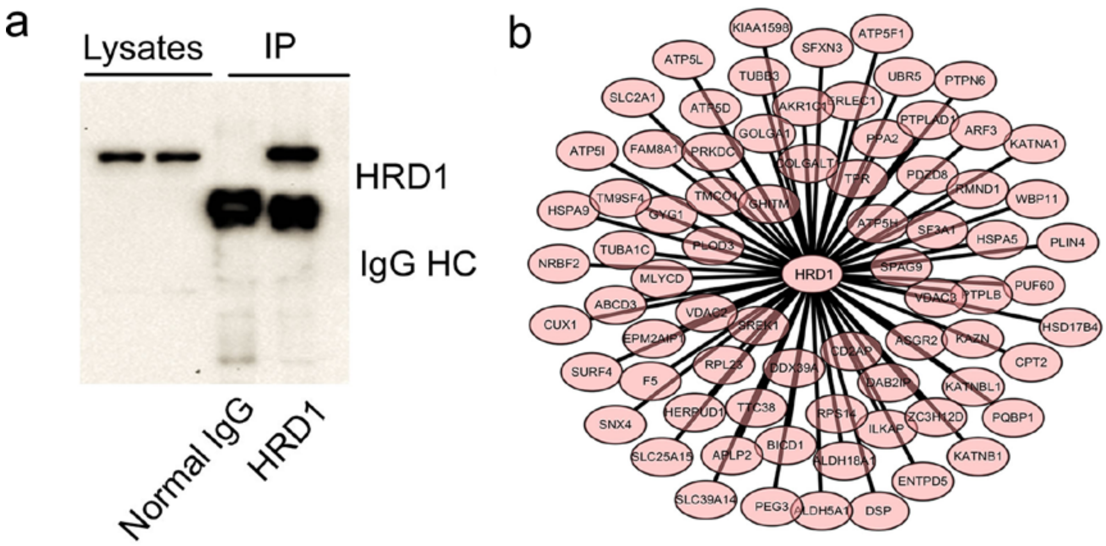

Supplementary Fig. 5 New HRD1 binding proteins screened by the AP-MS method. (**a**) Affinity purification of HRD1 complex. The successful purification of HRD1 complex was shown by Western-blot. Normal IgG was used as control. (**b**) New HRD1 binding proteins screened using the proteomic method.

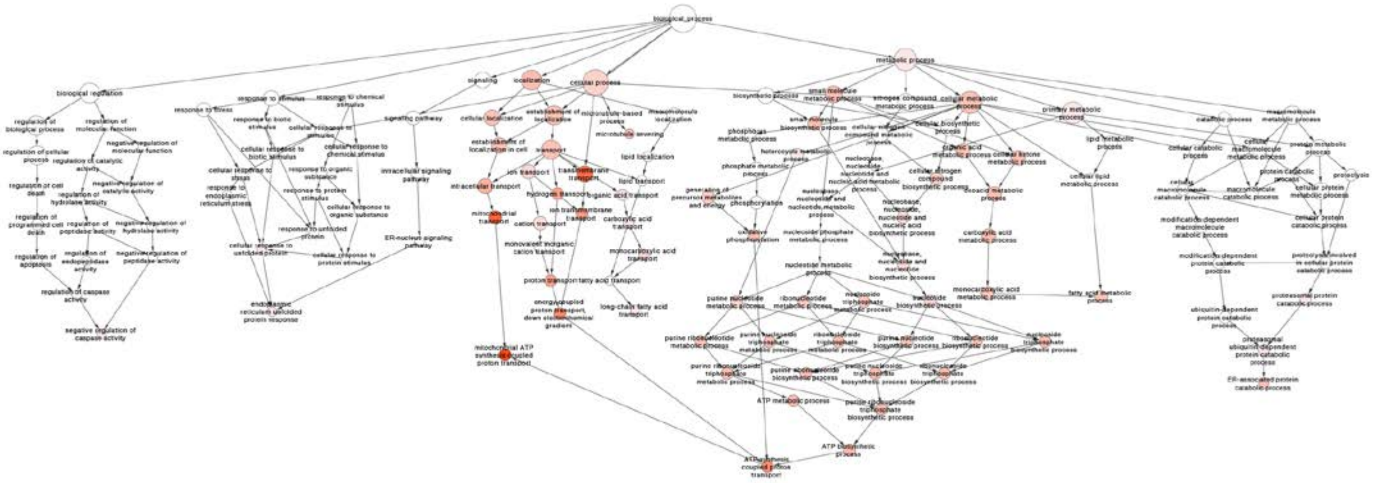

Supplementary Fig. 6: **Biological processes analysis of HRD1 interacting proteins.** The enrichment analysis was carried out by Cytoscape software with a Bingo plug-in. Hypergeometric test and Benjamini and Hochberg False Discover Rate correction was used. The enriched GO term was shown with dark red.

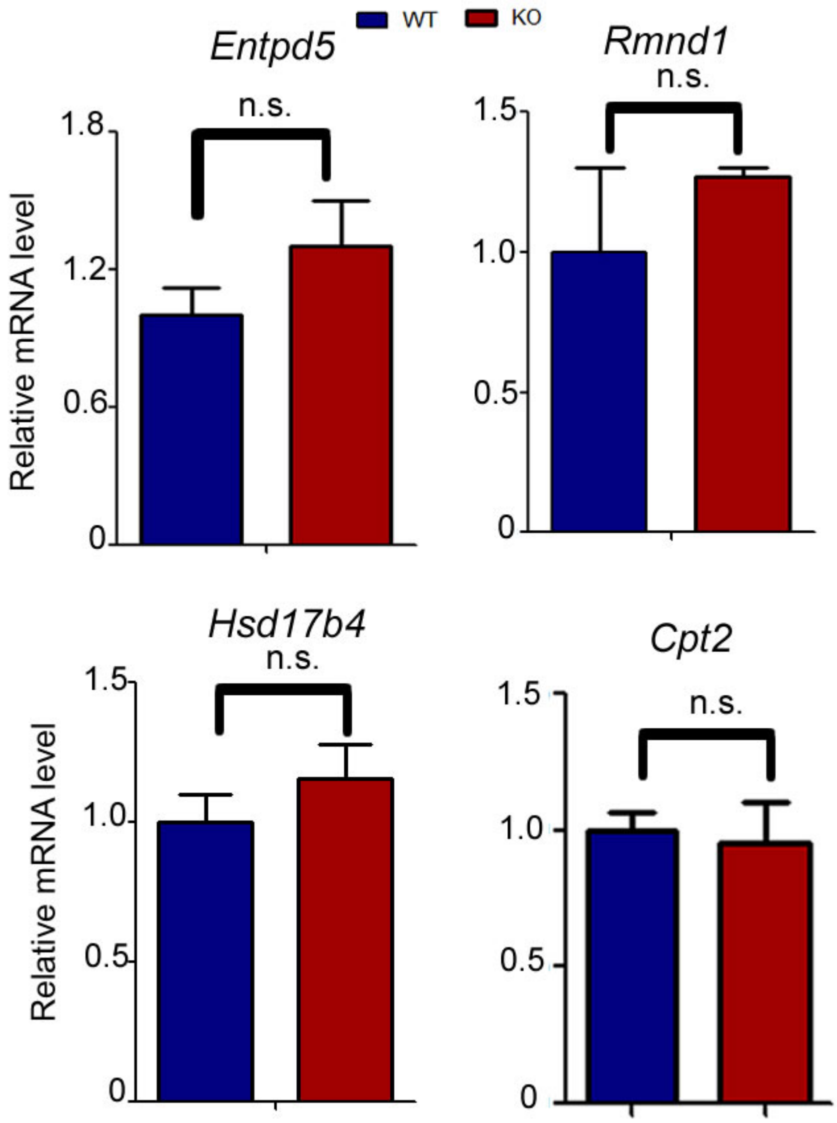

Supplementary Fig. 7 HRD1 deletion didn't change the mRNA level of Rmnd1, Entpd5 and Hsd17b4. Hepatic mRNA level of *Syvn1*, *Rmnd1*, *Entpd5* and *Hsd17b4* from WT and LKO mice. (n=5 for each group). The data are representative of three independent experiments (mean±SEM) *: $P<0.05$. **: $P<0.01$ by unpaired student's t test.

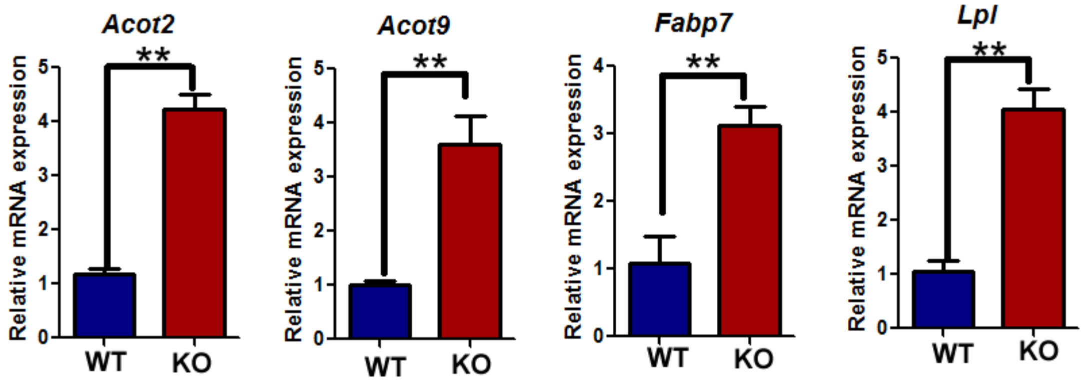

Supplementary Fig. 8 The genes involved in TG and fatty acid oxidation were increased after HRD1 deletion. Hepatic *Acot2, Acot9, Fabp7* and *Lpl* were measured from WT and HRD1 mice in a refed condition. (n=5 for each group). The data are representative of three independent experiments (mean±SEM) *: *P*<0.05. **: *P*<0.01 by unpaired student's t test.

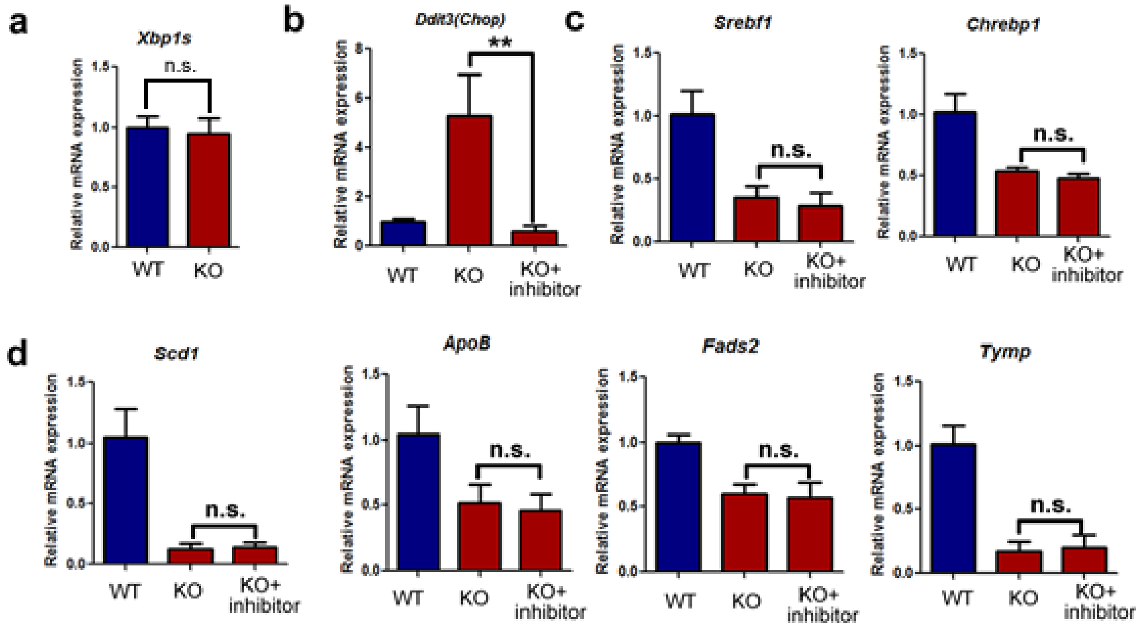

Supplementary Fig. 9 The effects of PERK inhibitor on metabolic responsive gene expression in HRD1 KO liver. (**a**) Hepatic Xbp1s were measured from WT and HRD1 mice in a refed condition. (**b-d**) WT and HRD1 LKO mice were fasted overnight and refed for 1 hour and then administrated with PERK inhibitor for additional 3 hours. Hepatic mRNA level of *Ddit3* (*Chop*) (b), *Srebf1* and *Chrebp1*(c) *Scd1*, *ApoB*, *Fads2* and *Tymp* (d) were measured. (n=5 for each group). The data are representative of three independent experiments (mean±SEM) *: *P*<0.05. **: *P*<0.01 by unpaired student's t test.

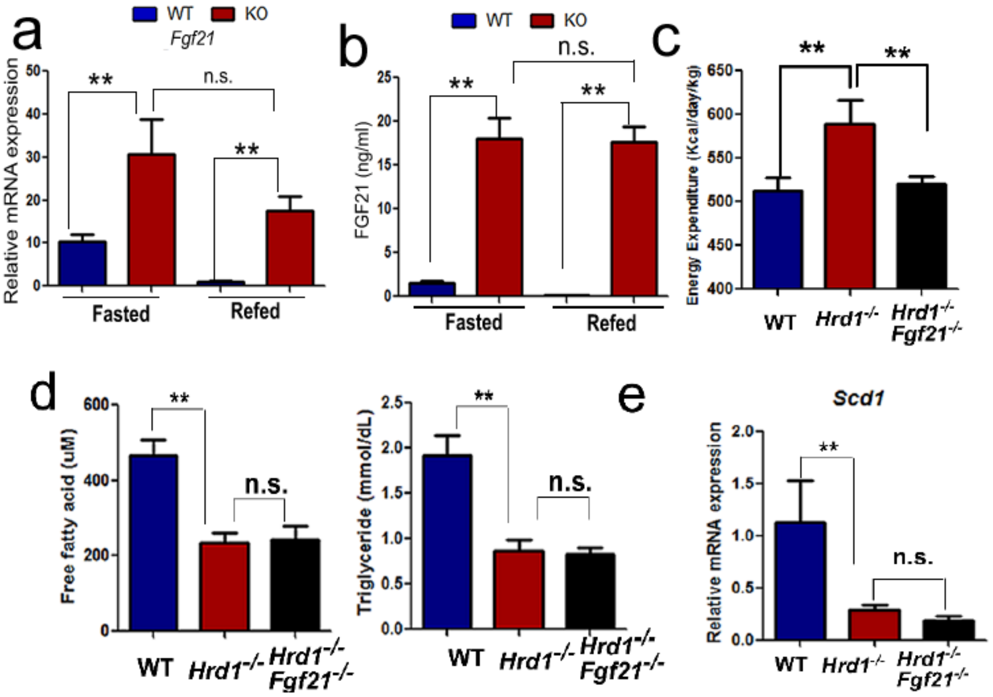

Supplementary Fig. 10. Elevation of energy expenditure of HRD1 LKO mice through FGF21 overexpression. (a) Hepatic Fgf21 mRNA in the WT and L-HRD1 KO mice. (n=5 for each group). **(b)** Serum FGF21 protein levels in the WT and L-HRD1 KO mice. (n=5 for each group). **(c)** Energy expenditure of WT, HRD1 LKO and HRD1/FGF21 DKO mice. (n=5 for each group). **(d)** Serum fatty acid and TG levels of WT, HRD1 LKO and HRD1/FGF21 DKO mice. (n=5 for each group). **(e)** Relative Scd1 mRNA expression of WT, HRD1 LKO and HRD1/FGF21 DKO mice. (n=5 for each group). The data are representative of three independent experiments (mean±SEM) *: $P<0.05$. **: $P<0.01$ by unpaired student's t test.

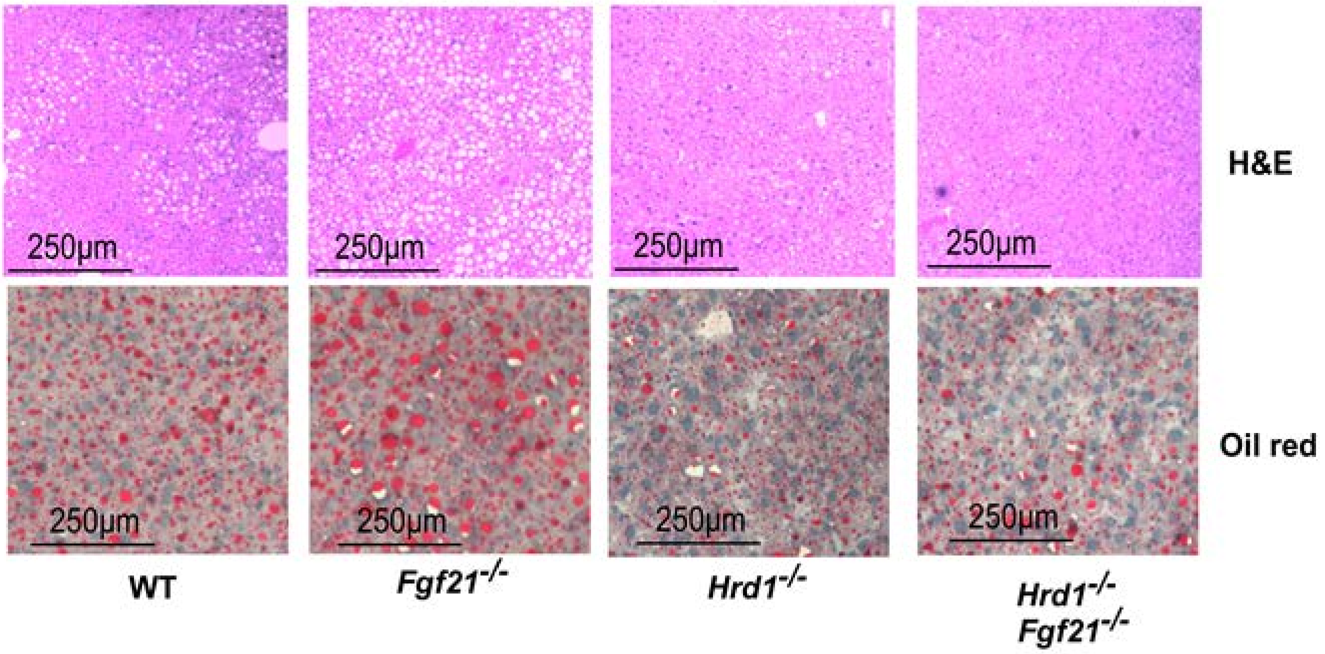

Supplementary Fig. 11. Reduction of lipid accumulation in HRD1 LKO mice was not through FGF21 overexpression. H&E (top panels) and Oil Red O (bottom panels) staining of the WT, Fgf21$^{-/-}$, HRD1$^{-/-}$ and Fgf21$^{-/-}$HRD1$^{-/-}$ livers after 14 weeks HFD treatment.

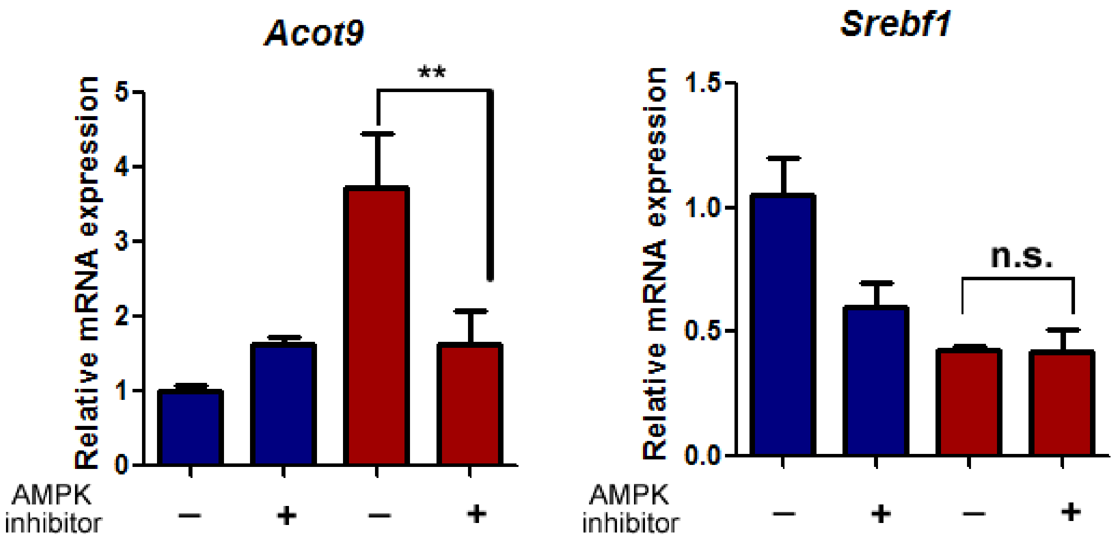

Supplementary Fig. 12 Effect of AMPK inhibitor on the gene expression in HRD1-null liver cells. WT and HRD1 LKO mice were fasted overnight and refed for 1 hour and then administrated with AMPK inhibitor for additional 3 hours. Hepatic mRNA level of *Acot9* and *Srebf1* were measured. (n=5 for each group). The data are representative of three independent experiments (mean±SEM) *: *P*<0.05. **: *P*<0.01 by unpaired student's t test.

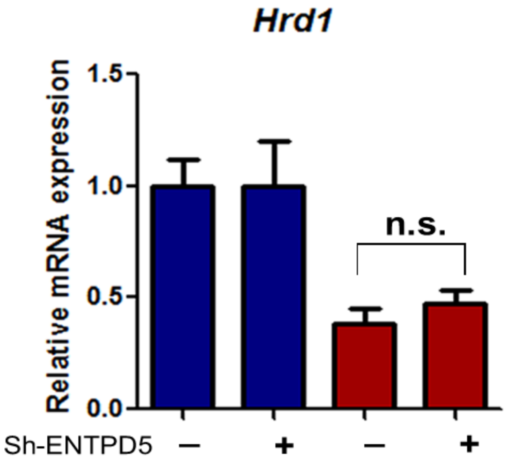

Supplementary Fig. 13 Effects of ENTPD5 knockdown on metabolic gene expression in HRD1-null hepatocytes. WT and HRD1 LKO hepatocytes were infected with lentivirus to specific knockdown *Entpd5*. 2 days after infection, Hepatic mRNA level *Hrd1* was measured. (n=5 for each group). The data are representative of three independent experiments (mean±SEM) *: $P<0.05$. **: $P<0.01$ by unpaired student's t test.

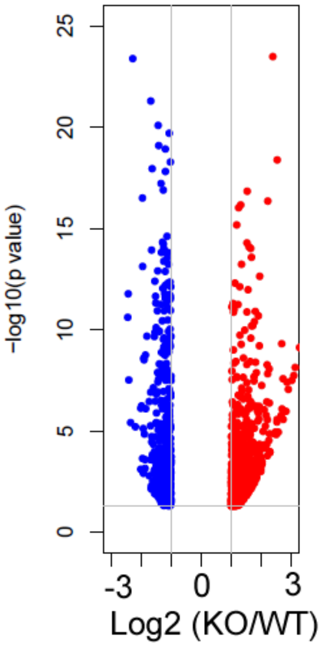

Supplementary Fig. 14 HRD1 was postprandially induced to control the metabolic balance in the physiological condition. Volcano plot of the differential genes from only in the refed condition.

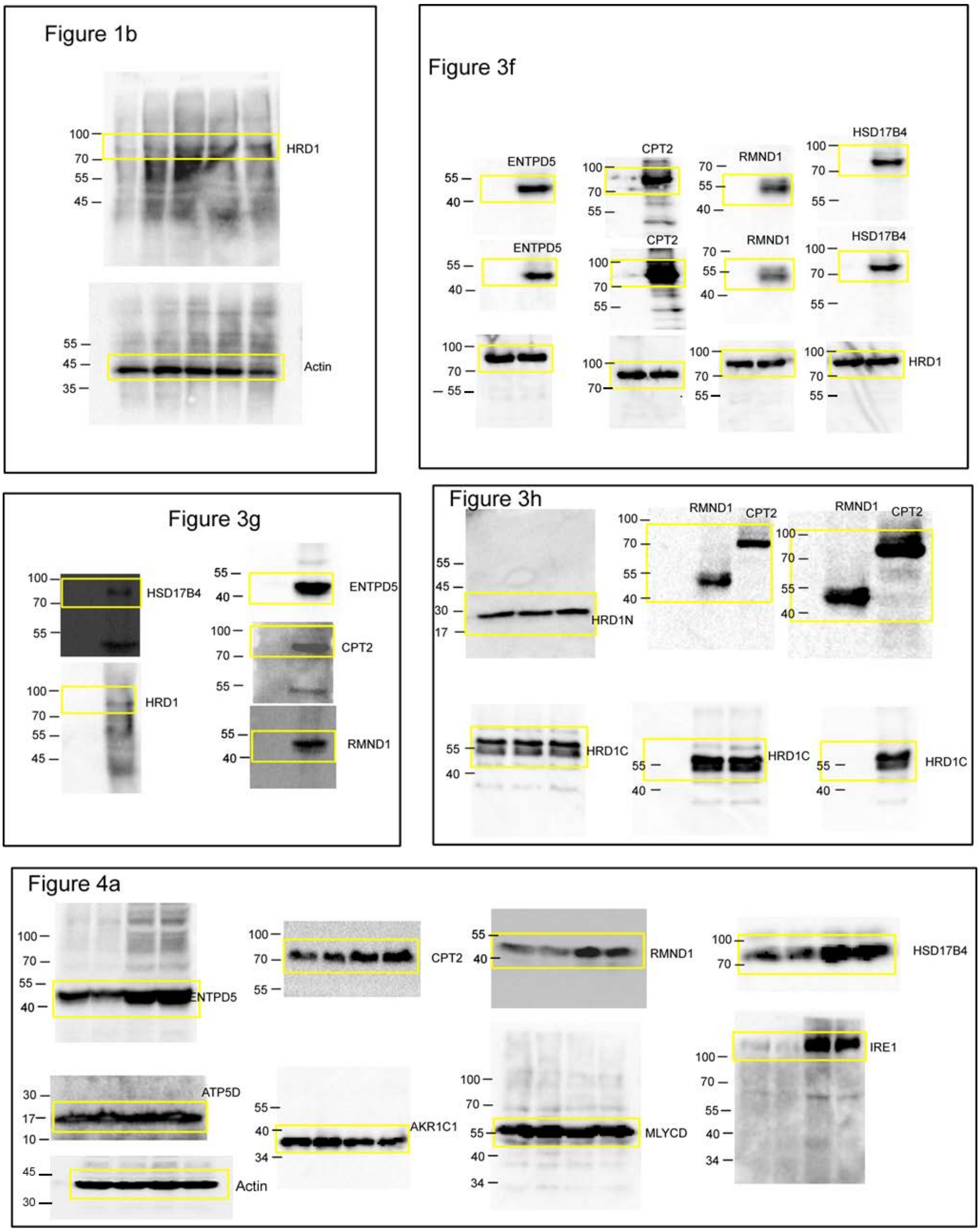

Supplementary Fig. 15 Uncropped blots for Fig. 1b-Fig. 4a. Note that yellow dashed represents the cropped image.

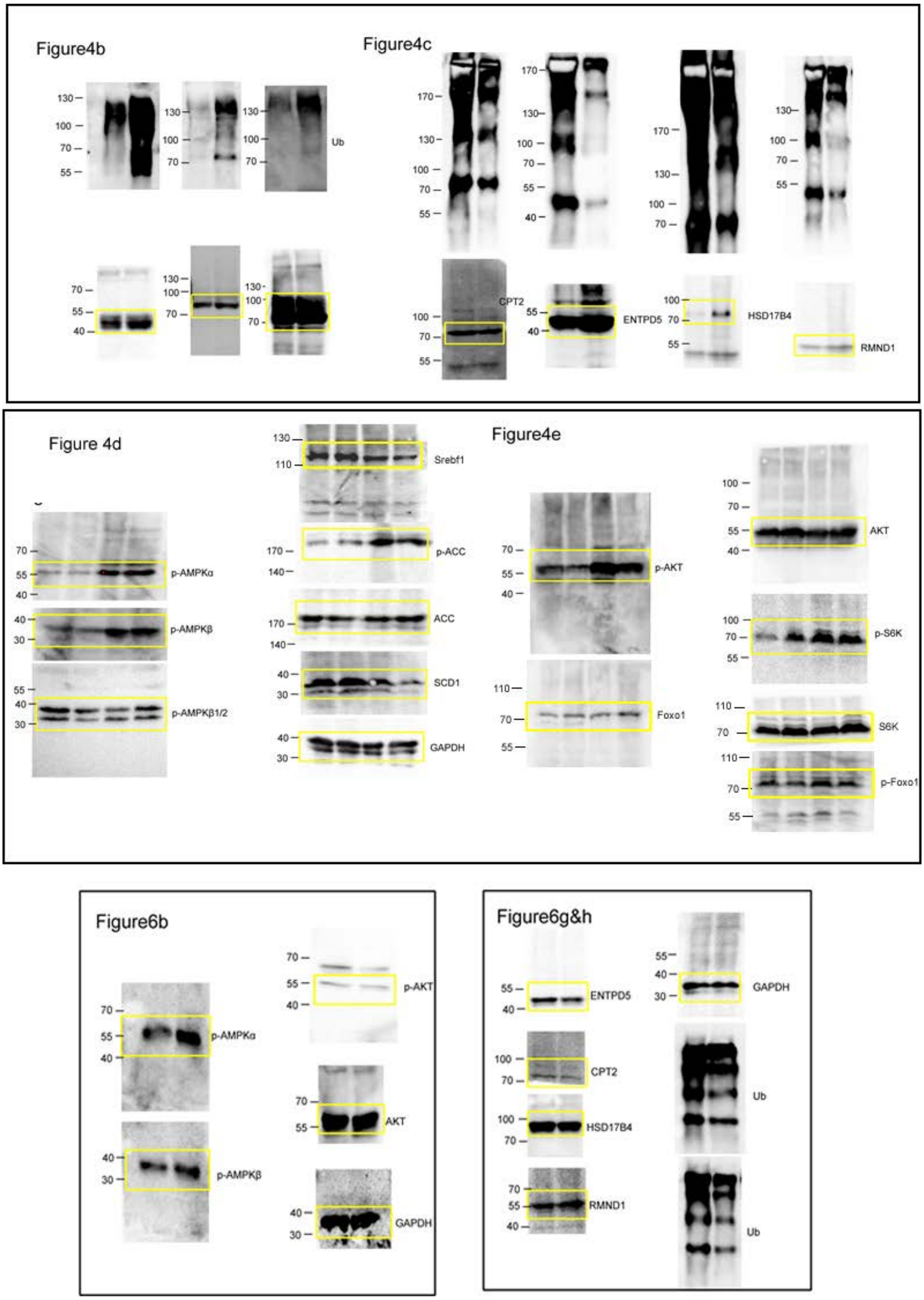

Supplementary Fig. 16 Uncropped blots for Fig. 4b-Fig. 6h. Note that yellow dashed represents the cropped image.
